# Semi-empirical model to estimate ideal conditions for the growth of large protein crystals

**DOI:** 10.1107/S205979832001445X

**Published:** 2020-11-26

**Authors:** Hirohiko Nakamura, Sachiko Takahashi, Koji Inaka, Hiroaki Tanaka

**Affiliations:** a Confocal Science Inc., Hayakawa 2nd Building 7F, 2-12-2 Iwamoto-cho, Chiyoda-ku, Tokyo 101-0032, Japan; b Maruwa Foods and Biosciences Inc., 170-1 Tsutsui-cho, Yamatokoriyama, Nara 639-1123, Japan

**Keywords:** large protein crystals, nucleation, crystal growth, crystallography, neutron protein crystallography, neutron diffraction, semi-empirical models, numerical models

## Abstract

A large high-quality crystal is required to specify the positions of H atoms in neutron structural analysis. Consequently, several methods have been proposed for obtaining such large crystals, and theoretical considerations for growing them have been presented. However, further investigation is required to obtain a numerical model that can provide quantitative experimental conditions for obtaining a single large crystal. In the case of protein crystallization experiments, the amount of sample is often limited. Therefore, it is more realistic to make a rough estimation from a small number of experiments. This paper proposes a method of estimating the optimum experimental conditions for the growth of large protein crystals by performing a small number of experiments using a micro-batch method and reporting a numerical model based on nucleation theory and a linear approximation of the crystal-growth rate. Specifically, micro-batch experiments are performed to provide the empirical parameters for the model and to help to estimate the conditions for the growth of a crystal of a predetermined size using a certain sample concentration and volume. This method is offered as a step on the path towards efficiently and rationally producing large crystals that can be subjected to neutron diffraction without depending on luck or on performing many experiments. It is expected to contribute to drug design and the elucidation of protein molecular functions and mechanisms by obtaining positional information on H atoms in the protein molecule, which is an advantage of neutron diffraction.

## Abstract 2

A semi-empirical model to estimate the optimum conditions for the growth of large protein crystals has been developed.

## 1. Introduction   

Neutron protein crystallography, a powerful neutron diffraction technique for investigating protein chemistry, has elicited considerable interest among academics and pharmaceutical companies. This growing interest stems from the building of new and improved beamlines, the development of improved software and the availability of novel techniques for growing larger crystals (Blakeley *et al.*, 2004 ▸; Niimura & Podjarny, 2011 ▸). However, only 0.1% of the macromolecular structures deposited in the Research Collaboratory for Structural Bioinformatics Protein Data Bank (PDB) were determined using neutron diffraction (as of June, 2020). This is mainly because the neutron diffraction process requires much larger cubic crystals (∼1 mm^3^) than X-ray diffraction. Additionally, X-ray diffraction is applied more generally than neutron diffraction because it requires relatively small amounts of sample. Furthermore, X-ray diffraction beamlines are easier to access than neutron diffraction beamlines. Even neutron beamtime review committees are required to access X-ray diffraction data first (Helliwell, 2017 ▸). Neutron diffraction can provide complementary data to X-ray diffraction for the location of H atoms, since only the electronic density of H atoms is visible using X-rays. In some cases, neutron diffraction can provide insights into H atoms and hydration in protein crystal structures that is not available from X-ray diffraction alone (Meilleur *et al.*, 2006 ▸). A complement of X-ray and neutron diffraction in biological science can provide the most complete biological structure possible.

To obtain large crystals, many crystallization methods have been proposed, such as vapour-diffusion (Kelpšas *et al.*, 2019 ▸; Koruza *et al.*, 2019 ▸; Fukuda *et al.*, 2020 ▸), batch (Dajnowicz *et al.*, 2017 ▸), dialysis (Zeppezauer, 1971 ▸; Maeda *et al.*, 2004 ▸; Niimura & Podjarny, 2011 ▸) and counter-diffusion (Ng *et al.*, 2015 ▸; Schaffner *et al.*, 2017 ▸) methods. These methods are sometimes combined with micro-seeding and macro-seeding (Thaller *et al.*, 1985 ▸; Bergfors, 2003 ▸), protein feeding (Bergfors, 2003 ▸) or temperature control (Budayova-Spano *et al.*, 2007 ▸).

The establishment of a protein crystallization phase diagram can guide the growth of large crystals (McPherson, 1999 ▸; Chayen *et al.*, 2010 ▸; Niimura & Podjarny, 2011 ▸; Nakamura *et al.*, 2013 ▸; Rupp, 2015 ▸). Controlling the nucleation and crystal growth occurring in the metastable zone between the solubility and super-solubility curves in the phase diagram, where the nucleation probability is low and crystals grow, can result in the growth of one large crystal (Saridakis & Chayen, 2000 ▸; Budayova-Spano *et al.*, 2020 ▸). However, crystal growth is not based on quantitative optimization but on many experiments, making it time-consuming, with results that require a high consumption of protein samples. Occasionally, a large crystal is grown but cannot be reproduced. Furthermore, even using a phase diagram, it is difficult to control the number of crystals to one.

The nucleation process has been studied theoretically (Galkin & Vekilov, 2001 ▸), but it has not been applied to the problem of the growth of large crystals. Another theoretical consideration involves utilization of the free energy as a function of the charge on the protein molecule (Ng *et al.*, 2015 ▸). Ostwald ripening (Ostwald, 1897 ▸) applies a process in which a small crystal is absorbed by a larger stable crystal. This has also been formulated qualitatively using equations related to free energy (Ng *et al.*, 2015 ▸). However, further investigation is required to obtain a numerical method that can provide quantitative experimental conditions for obtaining one large crystal.

In this study, we developed a method for estimating the optimum experimental conditions for the growth of a large crystal using a certain volume of sample solution. This method uses a combination of a few preliminary micro-batch experiments and a numerical model. The numerical model is based on nucleation theory and a linear approximation of the crystal-growth rate. The micro-batch experiments provide the empirical parameters for the model, and differential equations based on these parameters help to estimate the ideal conditions for the growth of a large single crystal in a certain sample volume. Thus, more efficient and rational crystallization experiments can be implemented to grow a single large protein crystal. In this study, *Microsoft Excel* 2016 was used for the data analysis and all graphs.

## 2. Numerical model   

### 2.1. Sample volume   

It is important to have a rough idea of the amount of protein sample that is required to grow a large crystal. For simplicity, the crystal is assumed to be a cube of dimension *a* (mm). The number of protein molecules in the crystal is calculated using equation (1), where *V*_M_ is the Matthews coefficient and *M* is the molecular weight,
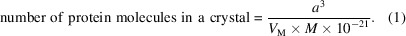
It is converted to moles using equation (2),
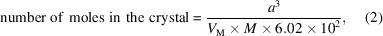
and to weight (mg) using equation (3), 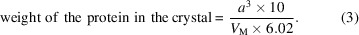
When the concentration of the protein sample solution is *C* (mg ml^−1^) and the solubility is *C*_e_ (mg ml^−1^), the required solution volume (µl) is expressed as
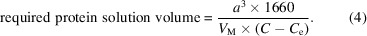



For example, in the case of lysozyme (PDB entry 3ijv; *V*_M_ = 1.84 Å^3^ Da^−1^; E. Pechkova, S. K. Tripathi & C. Nicolini, unpublished work), if the cubic crystal size is 1 mm^3^ then the sample solution concentration is 35 mg ml^−1^, the solubility is 4 mg ml^−1^ and the required amount of solution is approximately 29 µl. Thus, a rough estimation of the size of the crystallization container can be made before performing experiments.

It should be noted that *V*_M_ is an important index that is related to the solvent content and to the resolution of the diffraction data of the crystal. Generally, if *V*_M_ is smaller, the solvent volume in the crystal is smaller and the resolution of the crystal is higher (Kantardjieff & Rupp, 2003 ▸). The precision of the amount of protein needed and the volume required to grow a protein crystal of a certain size depends on the accuracy of *V*_M_.

### 2.2. Estimation of *C_e_* and *A*_1_   

It is known that protein crystal growth follows a linear differential equation (5) in the low super-saturation region, where *L*(*t*) is the characteristic length of the crystal at time *t* if the crystal is assumed to be a cube and *A*_1_ is a constant (Chernov, 1998 ▸),
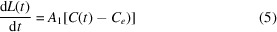



*A*_1_ is a parameter that is related to the bulk kinetic coefficient of crystal growth and is a constant which depends on the protein species, the type and concentration of the precipitant or additive, the pH, the environmental temperature *etc*.

**Figure 1:**
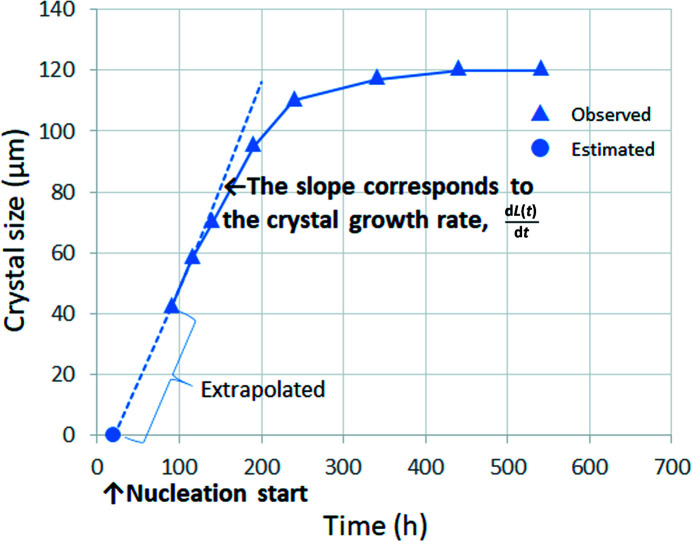
Estimation of the start time of nucleation and the initial crystal-growth rate. The initial crystal-growth rate d*L*(*t*)/d*t* can be extrapolated from the slope of an approximated straight line (dotted). The horizontal intercept of this line (circle) corresponds to the start of nucleation.

To estimate *C*_e_ and *A*_1_, crystallization experiments were performed under at least two conditions by changing the protein concentration. The time course for crystal growth was recorded from the beginning of crystal growth. Fig. 1 ▸ shows a plot of time on the horizontal axis versus crystal size on the vertical axis. The initial crystal-growth rate can be extrapolated using a straight approximated line with a slope corresponding to the crystal-growth rate d*L*(*t*)/d*t*. The horizontal intercept of this approximated straight line corresponds to the time when the nucleation started.

**Figure 2:**
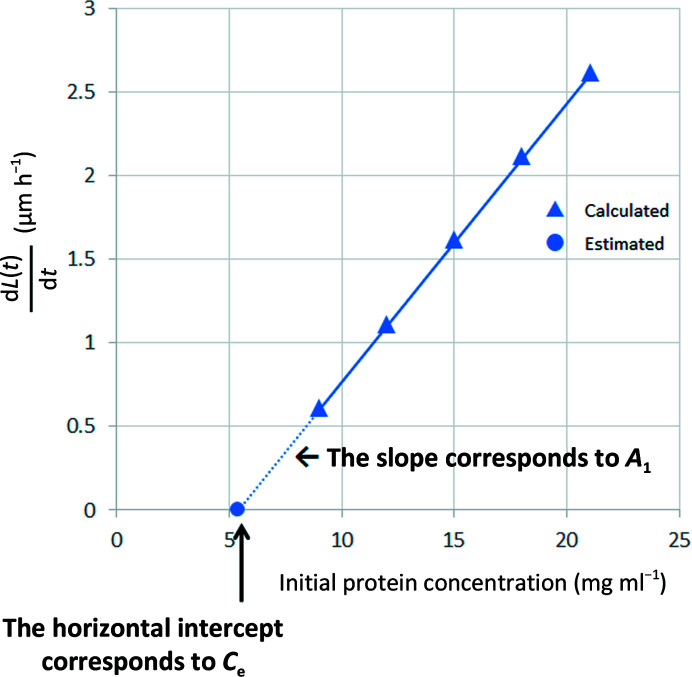
Estimation of *C*_e_ and *A*_1_. The horizontal intercept of this straight line (circle) corresponds to *C*_e_ and the slope corresponds to *A*_1_.

Next, the initial protein concentration on the horizontal axis was plotted against d*L*(*t*)/d*t* as obtained in Fig. 1 ▸ on the vertical axis (Fig. 2 ▸). At low protein concentrations, the plots were almost linear. The horizontal intercept of this straight line corresponds to *C*_e_ and its slope corresponds to *A*_1_ from equation (5).

### 2.3. Estimation of *A*_2_ and *A*_3_   

According to nucleation theory (Galkin & Vekilov, 2001 ▸; Yoshizaki *et al.*, 2002 ▸; García-Ruiz, 2003 ▸), the nucleation probability *I*(*t*) per unit time and unit volume at time *t* is explained by the following equation, where *C*(*t*) is the protein concentration in the solution at time *t* and *A*_2_ and *A*_3_ are constants,
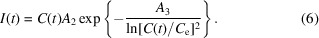



*A*_2_ is related to the frequency of the attachment of molecules to the critical size of the nucleus and *A*_3_ is related to the thermodynamic barriers to the creation of critical and spherical clusters (Galkin & Vekilov, 2001 ▸). Both parameters are assumed to be constant and depend on the protein species, the type and concentration of the precipitant or additive, the pH, the environmental temperature *etc*.

**Figure 3:**
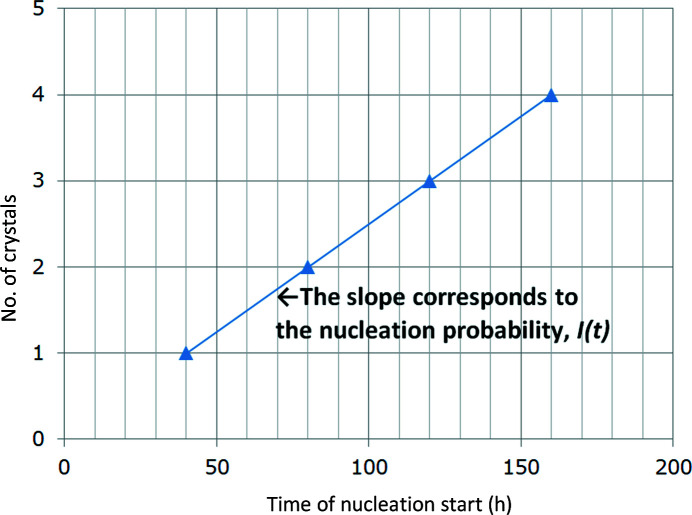
Estimation of the nucleation probability *I*(*t*) using the slope of the approximated straight line.

Generally, if the probability of nucleation is set to a unit volume at unit time, the number of crystals will increase with time in a larger volume of the solution. Therefore, if we plot the time for nucleation on the horizontal axis against the number of crystals on the vertical axis, *I*(*t*) can be calculated from the slope of the approximated straight line (Fig. 3 ▸).

**Figure 4:**
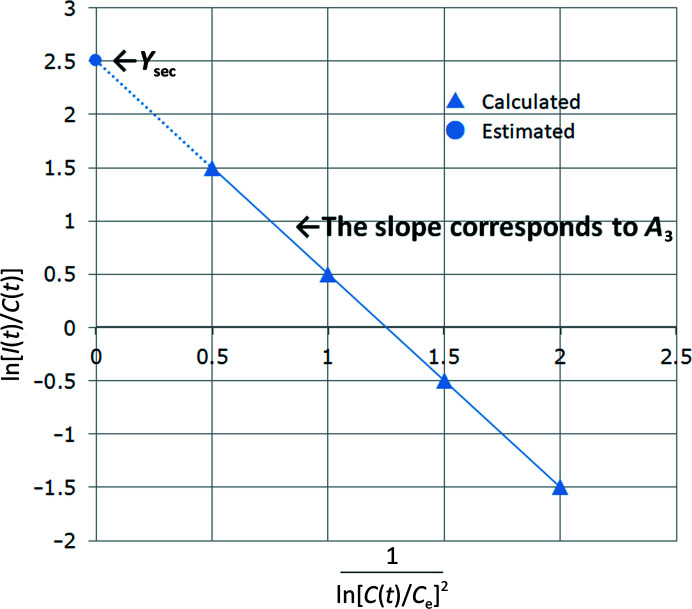
Estimation of *A*_2_ (*Y*_sec_) and *A*_3_. The vertical intercept of the straight line (circle) corresponds to *Y*_sec_ and the slope corresponds to *A_3_*.

Equation (6) can be rearranged to provide equation (7),
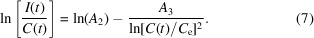



Therefore, by plotting 1/{ln[*C*(*t*)/*C*_e_]^2^} on the horizontal axis against ln[*I*(*t*)/*C*(*t*)] on the vertical axis and making a linear approximation, *A*_3_ can be estimated from the slope (Fig. 4 ▸). If the vertical intercept is substituted as *Y*_sec_, we obtain 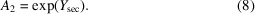



### 2.4. Crystallization experiment: prerequisites   

In the crystallization experiment, the protein concentration in the container decreases when a crystal starts to grow. Therefore, the second and subsequent crystals grow under different conditions to the first in the same container. Generally, movement of protein molecules in the container occurs because of thermal diffusion and density-driven convection (Nerad & Shlichta, 1986 ▸). However, density-driven convection is suppressed if the characteristic length of the container is small (García-Ruiz *et al.*, 2001 ▸). Therefore, we use a thin capillary placed horizontally to perform a micro-batch crystallization experiment. Thermal diffusion is a phenomenon in which a molecule moves owing to thermal fluctuations over time. If the fluctuations are one-dimensional, the molecules will move a distance calculated by the following equation, where *D* is the diffusion coefficient and is a unique value for the protein that depends on factors such as the molecular weight, shape and temperature, 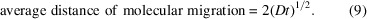



In the case of lysozyme, the diffusion coefficient is approximately 1.25 × 10^−10^ m^2^ s^−1^ in aqueous solution. However, in the case of a polymer solution containing 15% polyethylene glycol (PEG) 4000 the diffusion coefficient is approximately 0.5 × 10^−10^ m^2^ s^−1^, as estimated by the approximate equations of Tanaka *et al.* (2006 ▸). From equation (9), it will take approximately 135.2 h on average to migrate 10 mm. For example, in a 15% PEG 4000 solution, if the second crystal starts growing 10 mm from the first crystal in the same capillary more than 135.2 h after the first crystal grows, the second crystal will grow with a lower protein concentration than the first.

**Figure 5:**
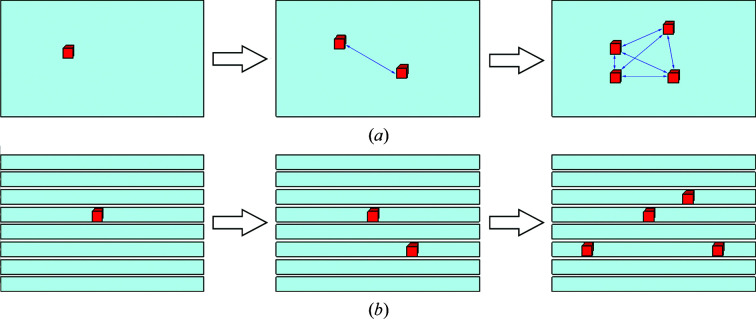
Many containers of small volume are suitable for measuring nucleation probability. (*a*) Appearance of crystals in a large-volume container: the nucleation of the second and subsequent crystals is affected by the first. (*b*) Appearance of crystals in small-volume containers: the total volume of these containers is the same as that in (*a*). The nucleation of the second and subsequent crystals is not affected by the first. A crystal that is sufficiently distant from other crystals in the same container is also not affected.

Therefore, to carry out micro-batch experiments to obtain parameters, the volume of each container should be reduced to target the first crystal in the capillary. To perform the required number of experiments, the number of capillaries can also be increased. To measure second and subsequent crystals that grow in the same container, the capillary should be sufficiently long and the second crystal should be separated by such a length that it is not affected by the first crystal (Fig. 5 ▸).

## 3. Experiments   

### 3.1. Micro-batch crystallization experiment   

The experimental conditions are shown in Table 1 ▸. Lysozyme (Confocal Science Inc., MB-P-AA001) was used in the crystallization experiments. Sodium chloride and sodium acetate trihydrate were obtained from FUJIFILM Wako Pure Chemical Corporation. Acetic acid and PEG4000 were obtained from Millipore Sigma. The only variable in the experiment was the protein concentration.

**Figure 6:**
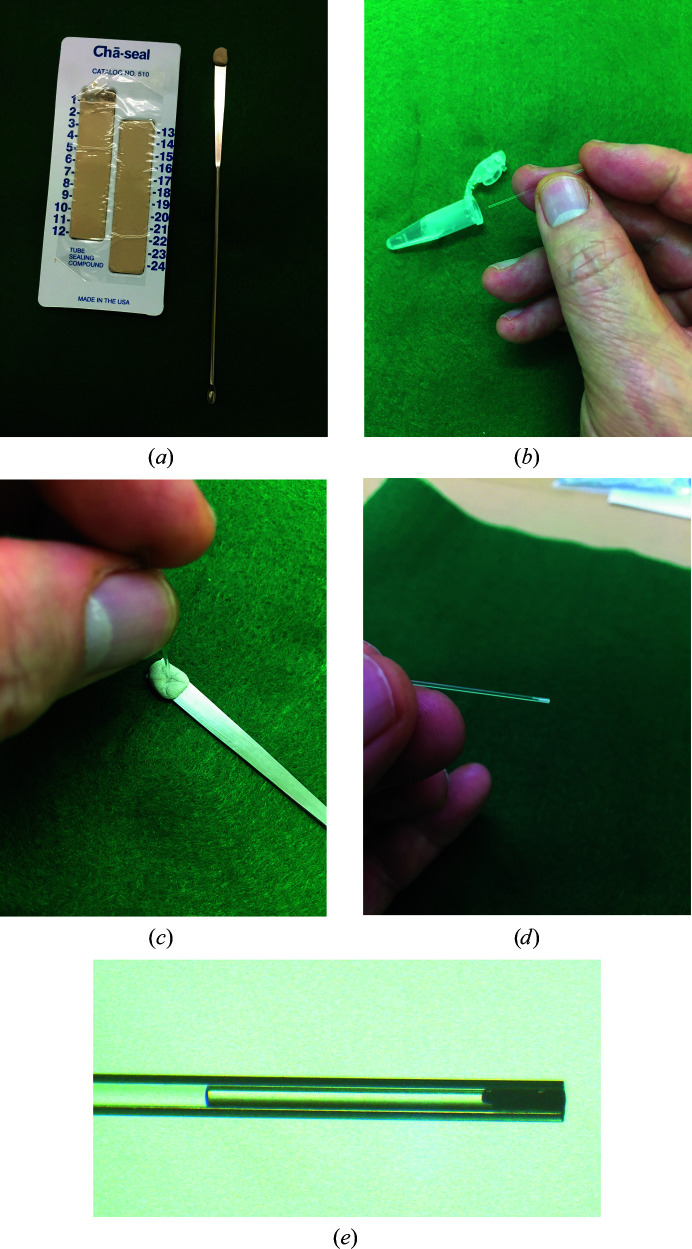
Micro-batch experimental procedure. (*a*) The sealing compound is attached to the flat part of a spatula. (*b*) The protein solution is loaded into a capillary. (*c*) Both ends of the capillary are sealed with the sealing compound. (*d*) The micro-batch crystallization cell is observed. (*e*) There should be a distance between the batch solution and the sealing compound because sometimes the sealing compound induces crystallization.

The micro-batch experiment was performed according to the following procedure. (i) Mix the batch solution to obtain the desired concentration and fill the capillary with it. Then seal both ends of the capillary with a sealing compound (Fig. 6 ▸). (ii) Periodically observe the inside of the capillary using a stereoscopic microscope (Nikon SMZ745) and record the images until the crystals stop growing. LED light is equipped in the microscope, so that no temperature change occurs during the observation of crystals.

### 3.2. Estimation of the parameters *C*_e_, *A*_1_, *A*_2_ and *A*_3_   

**Figure 7:**
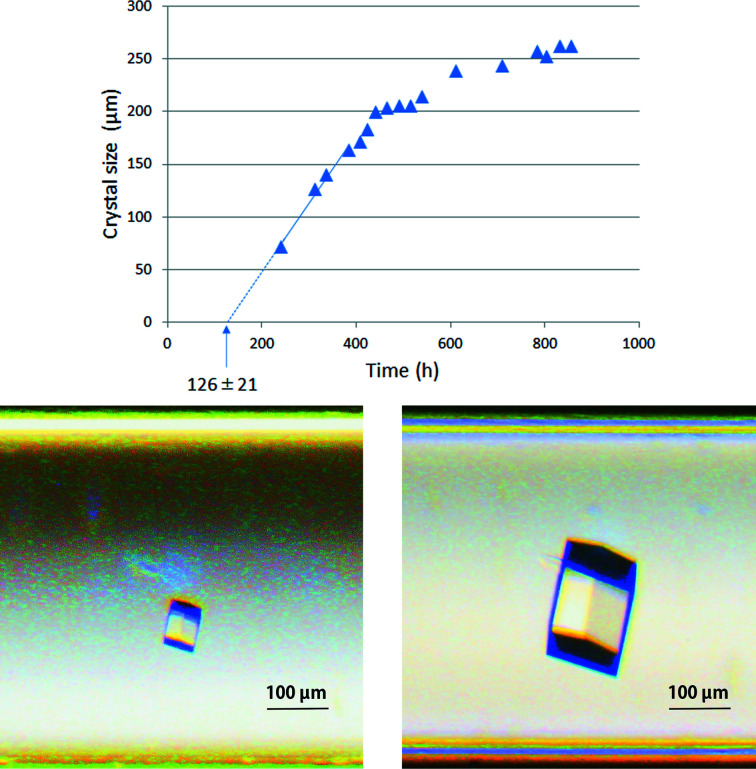
Time course of crystal growth. The batch solution volume was 2 µl 12 mg ml^−1^ lysozyme, 0.4 *M* sodium chloride, 15% PEG 4000, 0.04% sodium azide, 50 m*M* sodium acetate buffer pH 4.5. The initial crystal growth rate d*L*(*t*)/d*t* was extrapolated as 0.65 ± 0.06 µm h^−1^ based on the slope of the approximated straight line. The estimated nucleation start time was 126 ± 21 h from the horizontal intercept of this line. The photos are of the lysozyme crystal 241 h (left) and 855 h (right) after crystallization setup.

When a crystal was observed, its size was measured from the image data (Fig. 7 ▸). The crystal was approximated as a rectangular parallelepiped with aspect ratio *a*:*b*:*c* (see equation 11) and the length was measured in the longest direction. After the micro-batch experiment, some crystals were taken out, their lengths in three orthogonal directions were measured and their aspect ratios were calculated. Although they were relatively small crystals, we confirmed that the aspect ratio did not change significantly. It was assumed that the aspect ratio would not change much even if the size of the crystals was on a submillimetre scale.

Under the crystallization conditions listed in Table 1 ▸, one to three crystals were obtained from each container. Fig. 7 ▸ shows an example of crystal growth with 12 mg ml^−1^ lysozyme, 0.4 *M* sodium chloride, 15% PEG 4000, 0.04% sodium azide in 50 m*M* sodium acetate buffer pH 4.5. In accordance with the procedures described in Section 2.2 and Fig. 1 ▸, Fig. 7 ▸ was plotted. In this case, the nucleation start time was estimated to be 126 ± 21 h and the initial crystal-growth rate d*L*(*t*)/d*t* was estimated to be 0.65 ± 0.06 µm h^−1^. The same estimation was also performed under other conditions.

**Figure 8:**
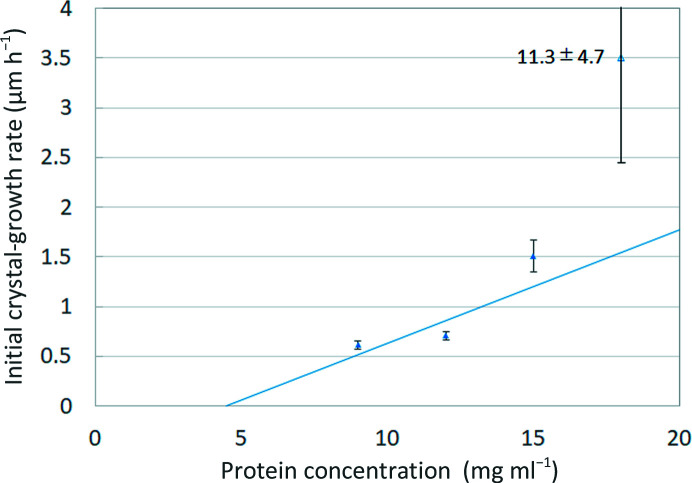
Estimation of *C_e_* and *A*_1_ from the experimental data. *C*_e_ was extrapolated to 4.51 ± 5.78 mg ml^−1^ from the horizontal intercept of the straight line and *A*_1_ was estimated to be 0.11 ± 0.05 µm ml mg^−1^ h^−1^ from the slope of this line. The growth rate from 18 mg ml^−1^ lysozyme solution was extremely fast and deviated from the straight line. The mode of crystal growth may differ for 15 and 18 mg ml^−1^ lysozyme solutions.

In accordance with the procedure described in Section 2.2 and Fig. 2 ▸, the protein concentration and the initial crystallization growth rate were plotted, as shown in Fig. 8 ▸, from which *C*_e_ and *A*_1_ were estimated (Table 2 ▸). *A*_1_ was estimated to be 0.11 ± 0.05 µm ml mg^−1^ h^−1^ from the slope of the straight line when the lysozyme concentration was below 15 mg ml^−1^ and *C*_e_ was estimated to be 4.51 ± 5.78 mg ml^−1^ from the horizontal intercept. The standard deviation of *C*_e_ seemed to be rather large. However, it depended on the estimated standard deviations of *A*_1_ and the vertical intercept point of equation (5). In Fig. 8 ▸, the estimation was based on the approximated straight line up to 15 mg ml^−1^ lysozyme solution. However, the growth rate from 18 mg ml^−1^ lysozyme solution was extremely fast and deviated from the straight line. The mode of crystal growth may differ for 15 and 18 mg ml^−1^ lysozyme solutions.

**Figure 9:**
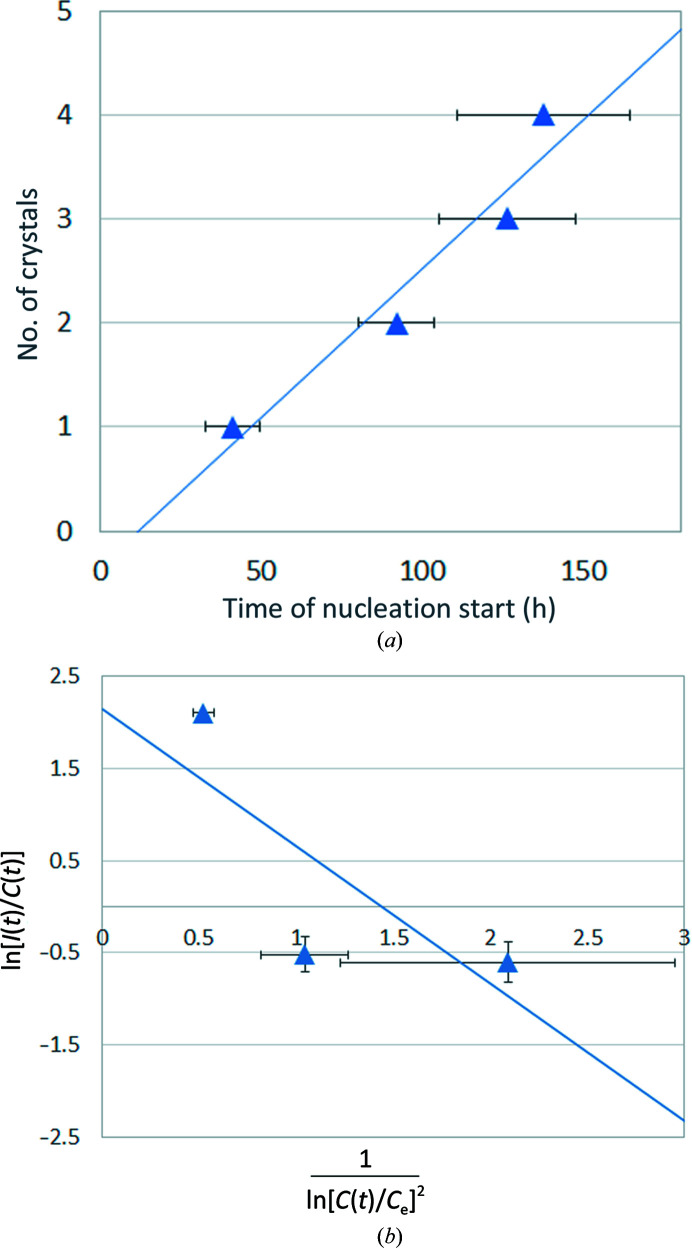
(*a*) Estimation of the nucleation probability *I*(*t*). This is an example of a plot when the lysozyme concentration was 12 mg ml^−1^, in which case *I*(*t*) was estimated to be 0.029 ± 0.006 h^−1^. (*b*) Estimations of *A*_2_ and *A*_3_ from the experimental data. *A*_2_ was calculated as 8.47 ± 5.37 by equation (8), using the vertical intercept of the straight line (*Y*_sec_), and *A*_3_ was estimated to be 1.49 ± 1.22 from the slope of this straight line.

In accordance with the procedure shown in Figs. 3 ▸ and 4 ▸, the plot shown in Fig. 9 ▸(*a*) was drawn to estimate the nucleation probability *I*(*t*) and Fig. 9 ▸(*b*) was drawn to estimate *A*_2_ and *A*_3_ (Table 2 ▸). Fig. 9 ▸(*a*) shows an example of the plot at a lysozyme concentration of 12 mg ml^−1^, in which *I*(*t*) was estimated to be 0.029 ± 0.006 h^−1^. The same calculation was also made under other conditions . In Fig. 9 ▸(*b*), *A*_2_ was calculated to be 8.47 ± 5.37 from the vertical intercept, and *A*_3_ was estimated to be 1.49 ± 1.22 from the slope of the straight line.

## 4. Results and discussion   

### 4.1. Comparison with experimental results   

**Figure 10:**
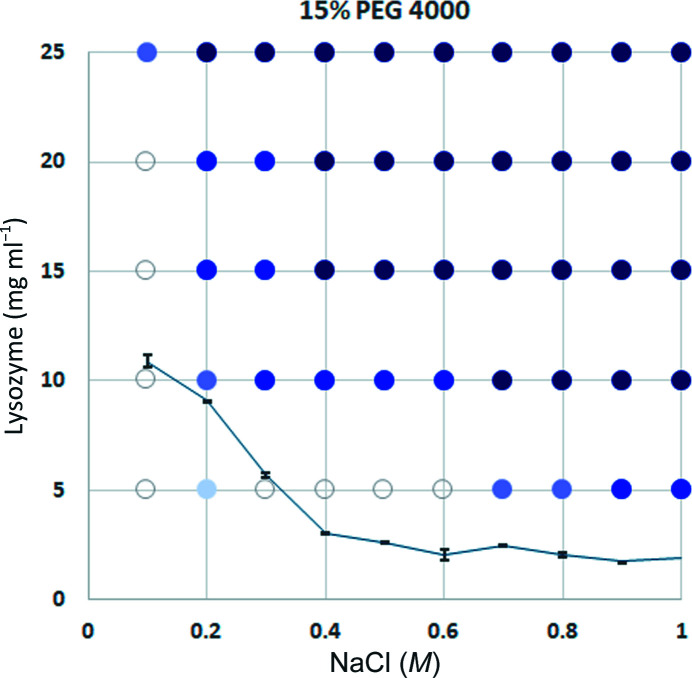
Phase diagram of lysozyme crystallization in 15% PEG 4000 with various concentrations of lysozyme and sodium chloride. The crystallization condition without lysozyme and sodium chloride was 15% polyethylene glycol, 0.04% sodium azide in 50 m*M* sodium acetate buffer pH 4.5 using the batch method. The darkest blue circles indicate crystals that appeared within 3 h of crystallization setup. Darker blue circles indicate those that appeared within three days. Light blue circles indicate those that appeared within seven days. Lighter blue circles indicate those that appeared after seven days. White circles indicate no crystal growth. The line indicates the solubility of lysozyme.

In our previous experiments, lysozyme at various concentrations was crystallized in 15% PEG 4000, 0.04% sodium azide in 50 m*M* sodium acetate buffer pH 4.5 with various concentrations of sodium chloride using a batch method. The solution volume was about 3 µl for each. Crystallization was observed until six months after the experimental setup, and the solubility of lysozyme was measured using the remaining solutions after removing crystals at the end of the experiment. The results are shown in Fig. 10 ▸. When the sodium chloride concentration was 0.4 *M*, which is the same condition as the experimental condition in Table 1 ▸, no crystals grew, even after six months, in 5 mg ml^−1^ lysozyme solution. However, in the case of 10 mg ml^−1^ lysozyme, seven crystals, with a size of about 0.15 mm, grew after 24 h. The solubility of lysozyme was 3.02 ± 0.09 mg ml^−1^, which was not so different from the estimated value of 4.51 ± 5.78 mg ml^−1^ in Table 2 ▸.

### 4.2. Prediction of nucleation probability   

**Figure 11:**
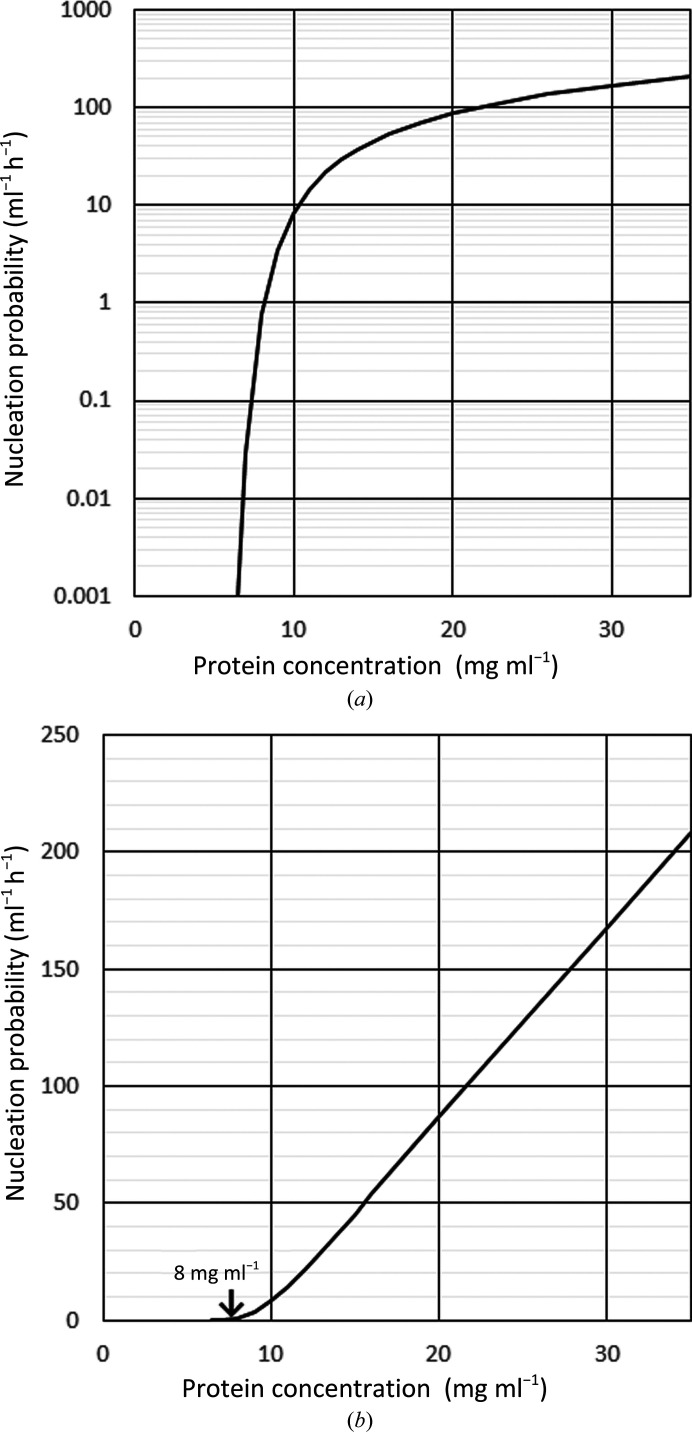
Nucleation probability depending on protein concentration. The vertical axis in (*a*) is a logarithmic scale in order to show where the nucleation probability is low. The vertical axis in (*b*) is a linear scale in order to show the border between the so-called metastable and nucleation zones.

By applying the parameters listed in Table 2 ▸ to equation (6), the nucleation probability *I* (in ml^−1^ h^−1^) for a protein concentration *C* (in mg ml^−1^) can be calculated as shown in Fig. 11 ▸. To show the slight difference in protein concentration in the low nucleation-probability range, the vertical axis in Fig. 11 ▸(*a*) is presented on a logarithmic scale. The nucleation probability increased rapidly up to 10 mg ml^−1^, which was approximately twice the solubility of the protein. However, at higher protein concentrations the rate of nucleation-probability increase gradually slowed. To show the critical protein concentration needed for crystal growth, the vertical axis in Fig. 11 ▸(*b*) is shown on a linear scale. This figure indicates that the border between the so-called metastable and nucleation zones in the phase diagram was around 8 mg ml^−1^.

### 4.3. Prediction of crystal number, crystal size and starting time for crystal growth   

**Figure 12:**
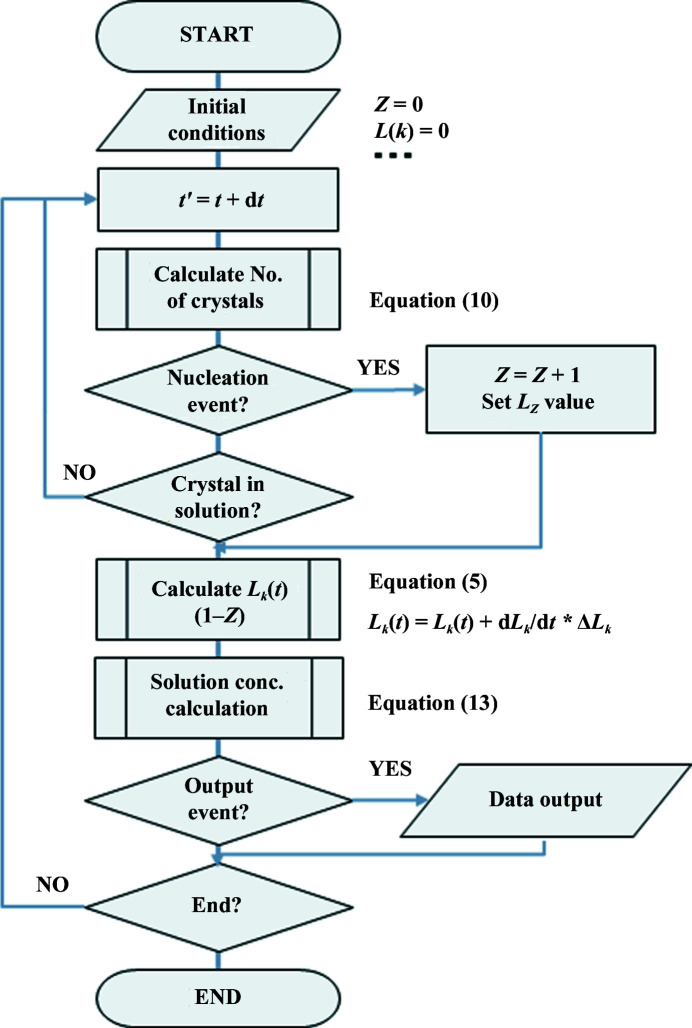
Flow chart of the calculation sequence.

Multiplying the nucleation probability by the container volume *V*, the expected increase in number of crystals per unit time is expressed by the following equation, where *N* is the number of crystals, 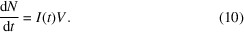



In the case of the growth of one crystal, the amount of increase in crystal volume Δ*V*_cryst_ in a small difference time Δ*t* is expressed by the following equation, where *L*(*t*) is one side length of the crystal and *a*, *b* and *c* are the aspect ratios of each side, which are 1.00, 0.84 and 0.77, based on the observation of the typical crystals:
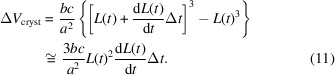



Because the amount of protein removed from the solution part in this small difference time is *C*_s_Δ*V*_cryst_, where *C*_s_ is the protein concentration in the crystal, equation (12) can be derived,
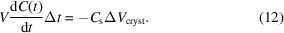



As the number of crystals increases and each crystal grows, the protein concentration change in the solution can be calculated by equation (13) if the protein concentration in the solution decreases uniformly. *Z* is the total number of crystals and *L*_*k*_(*t*) is one side length of the *k*th crystal,
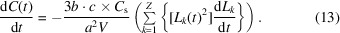



The actual crystal nucleation and its growth can be calculated using the sequence of equations (10), (5) and (13), as shown in the flow chart in Fig. 12 ▸. During calculation, *C*_s_ was 0.901 g ml^−1^ from equation (3), assuming a *V*_M_ of 1.84 Å^3^ Da^−1^.

By repeated calculation, the number of crystals and the final size of the *k*th crystal can be estimated. The number and size of the crystals and the starting time of crystal growth were calculated and are compared with our previous experimental results in Table 3 ▸. The experimental and calculated results were almost consistent. Thus, it can be said that the semi-empirical model can predict the crystal-growth process.

### 4.4. Growth of a large crystal   

To obtain only one large crystal, considering the results in Table 3 ▸ and Fig. 11 ▸(*b*), it seems to be necessary to reduce the number of crystals while increasing the crystal size. However, it is difficult to experimentally find the optimum protein concentration and the solution volume. When using the semi-empirical model, the number, crystal size and the starting time of crystal growth can be predicted finely with an initial protein concentration of lower than 10 mg ml^−1^. Thus, it is possible to evaluate the conditions in which the size of the first crystal exceeds 1 mm^3^ while changing the solution volume and protein-solution concentration (Table 4 ▸).

For example, if the solution volume was 400 µl and the initial protein concentration was 6.57 mg ml^−1^, the first crystals begin to grow after 1600 h, the protein sample concentration in the container decreases and the second crystal does not grow. It should be noted that the protein concentration is significantly lower than the border between the so-called metastable and nucleation zones in the phase diagram, which is around 8 mg ml^−1^ (Fig. 11 ▸ *b*). It is shown that the number of crystals and the initiation time for crystal growth are sensitive to the initial protein concentration. As summarized in Table 4 ▸, a concentration difference of 0.1 mg ml^−1^ or less makes a large difference in the time required and the number of crystals that begin growth. Therefore, when growing one large crystal using the batch method, one should set the initial experimental conditions carefully, paying particular attention to slight differences in protein concentrations, such as those of 0.1 mg ml^−1^, and a precise concentration study is necessary for the final stage of optimization of the crystallization condition.

## 5. Conclusions   

This paper proposes a semi-empirical model to estimate the optimum conditions for the growth of large protein crystals. The four parameters necessary for modelling the nucleation and crystallization process were obtained by performing crystallization experiments using the micro-batch method with different protein concentrations.

Using these parameters, we calculated the protein concentration and the amount of solution required to grow a single crystal of a predetermined size. As shown in Table 4 ▸, when the batch solution was 6.57 mg ml^−1^ lysozyme in a volume of 400 µl only one cubic crystal appeared with a size of 1.087 mm, which began nucleation after 1600 h. However, the number of crystals increased to ten when the protein concentration was increased by 5%. Therefore, the next challenge is to perform a precise concentration study to enable the growth of only one large crystal. This means that the traditional phase-diagram approach may not easily find the optimum protein concentration, which is much lower than the border between the so-called metastable area and the nucleation area in the batch method.

In our study, the number of experiments was found to be insufficient and the accuracy of the four parameters could have been improved by increasing the number of experiments. Proteins other than lysozyme should be applied in future experiments to expand the scope of this simulation.

Furthermore, only the protein concentration was changed in the crystallization conditions in this study. However, changing the concentrations of other components (for example sodium chloride and/or PEG) using other crystallization methods could create conditions under which large crystals would easily grow. The method introduced in this study can be applied to optimize the conditions in a wider range of crystallization conditions. Future studies could also evaluate how to change the abovementioned four parameters *C*_e_, *A*_1_, *A*_2_ and *A*_3_. Moreover, the method presented in this study can also be applied to the design of crystallization conditions for X-ray crystallography.

Because nucleation is a stochastic process, researchers should incorporate the standard deviation for multiple experiments. However, in the case of protein crystallization, the amount of sample is often limited. Therefore, it is more realistic to make a rough estimation from a smaller number of experiments. Applying the method introduced in this study would be a step on the path towards efficiently and rationally producing large crystals that can be subjected to neutron diffraction without depending on luck or on performing many experiments. We expect that this work will contribute to drug design and the elucidation of the molecular functions and biological mechanisms of proteins by obtaining positional information on H atoms in protein molecules, which is an advantage of neutron diffraction.

## Figures and Tables

**Table 1:** Experimental conditions

Lysozyme concentrations (mg ml^−1^)	9, 12, 15, 18
Precipitant, additives and buffer	0.4 *M* sodium chloride, 15% PEG 4000, 0.04% sodium azide, 50 m*M* sodium acetate buffer pH 4.5
Solution volume	4 µl (2 µl × 2)
Crystallization container	Two capillaries with an inner diameter of 0.5 mm and a length of 40 mm

**Table 2:** Estimated parameters

*C*_e_ (mg ml^−1^)	4.51 ± 5.78
*A*_1_ (µm ml mg^−1^ h^−1^)	0.11 ± 0.05
*A*_2_	8.47 ± 5.37
*A*_3_	1.49 ± 1.22

**Table 3:** Experimental and calculated results for crystal numbers, crystal size and initiation time of crystal growth

Initial protein concentration (mg ml^−1^)	5	10	15	20	25
Amount of solution (µl)	3.0	3.1	1.7	2.5	3.5
Experimental results†					
No. of crystals	0	7	3	11	34
Initiation time of crystal growth (h)	N/A	24	1–3	1–3	1–3
Calculated results					
No. of crystals	0	6	10	21	36
First crystal size (µm)	N/A	223	202	213	224
Initiation time of crystal growth (h)	>6 months	39.6	13	4.6	2.2

† The obtained crystals were rectangular parallelepipeds of 150–250 µm in length.

**Table 4:** The initial protein concentration affects the number of crystals and the initiation time of crystal growth The solution volume is 400 µl.

Initial protein concentration (mg ml^−1^)	Size of first crystal (mm)	No. of crystals	Initiation time of crystal growth (h)
6.55	1.12	1	1900
6.56	1.12	1	1800
6.57	1.09	1	1600
6.58	1.06	2	1500
6.6	1.03	2	1250
6.8	0.82	5	300
7.0	0.66	12	100

## References

[bb1] Bergfors, T. (2003). *J. Struct. Biol.* **142**, 66–76. 10.1016/s1047-8477(03)00039-x 12718920

[bb2] Blakeley, M. P., Cianci, M., Helliwell, J. R. & Rizkallah, P. J. (2004). *Chem. Soc. Rev.* **33**, 548–557. 10.1039/b312779f 15480478

[bb3] Budayova-Spano, M., Dauvergne, F., Audiffren, M., Bactivelane, T. & Cusack, S. (2007). *Acta Cryst.* D**63**, 339–347. 10.1107/S0907444906054230 17327671

[bb4] Budayova-Spano, M., Koruza, K. & Fisher, Z. (2020). *Methods Enzymol.* **634**, 22–46. 10.1016/bs.mie.2019.11.015 32093834

[bb5] Chayen, N. E., Helliwell, J. R. & Snell, E. H. (2010). *Macromolecular Crystallization and Crystal Perfection*. Oxford University Press.

[bb6] Chernov, A. A. (1998). *Acta Cryst.* A**54**, 859–872. 10.1107/s0108767398008587 9859196

[bb7] Dajnowicz, S., Johnston, R. C., Parks, J. M., Blakeley, M. P., Keen, D. A., Weiss, K. L., Gerlits, O., Kovalevsky, A. & Mueser, T. C. (2017). *Nat. Commun.* **8**, 955. 10.1038/s41467-017-01060-y PMC5643538 29038582

[bb8] Fukuda, Y., Hirano, Y., Kusaka, K., Inoue, T. & Tamada, T. (2020). *Proc. Natl Acad. Sci. USA*, **117**, 4071–4077. 10.1073/pnas.1918125117 PMC7049163 32041886

[bb9] Galkin, O. & Vekilov, P. G. (2001). *J. Cryst. Growth*, **232**, 63–76.

[bb10] García-Ruiz, J. M., Novella, M. L., Moreno, R. & Gavira, J. A. (2001). *J. Cryst. Growth*, **232**, 165–172.

[bb11] García-Ruiz, J. M. (2003). *J. Struct. Biol.* **142**, 22–31. 10.1016/s1047-8477(03)00052-2 12718933

[bb12] Helliwell, J. R. (2017). *Biosci. Rep.* **37**, BSR20170204. 10.1042/BSR20170204 PMC6434086 28572170

[bb13] Kantardjieff, K. A. & Rupp, B. (2003). *Protein Sci.* **12**, 1865–1871. 10.1110/ps.0350503 PMC2323984 12930986

[bb14] Kelpšas, V., Lafumat, B., Blakeley, M. P., Coquelle, N., Oksanen, E. & von Wachenfeldt, C. (2019). *Acta Cryst.* F**75**, 260–269. 10.1107/S2053230X19001882 PMC6450519 30950827

[bb15] Koruza, K., Mahon, B. P., Blakeley, M. P., Ostermann, A., Schrader, T. E., McKenna, R., Knecht, W. & Fisher, S. Z. (2019). *J. Struct. Biol.* **205**, 147–154. 10.1016/j.jsb.2018.12.009 30639924

[bb16] Maeda, M., Chatake, T., Tanaka, I., Ostermann, A. & Niimura, N. (2004). *J. Synchrotron Rad.* **11**, 41–44. 10.1107/s0909049503023859 14646130

[bb17] McPherson, A. (1999). *Crystallization of Biological Macromolecules*. New York: Cold Spring Harbor Laboratory Press.

[bb18] Meilleur, F., Myles, D. A. A. & Blakeley, M. P. (2006). *Eur. Biophys. J.* **35**, 611–620. 10.1007/s00249-006-0074-6 16897039

[bb19] Nakamura, A., Ishida, T., Fushinobu, S., Kusaka, K., Tanaka, I., Inaka, K., Higuchi, Y., Masaki, M., Ohta, K., Kaneko, S., Niimura, N., Igarashi, K. & Samajima, M. (2013). *J. Synchrotron Rad.* **20**, 859–863. 10.1107/S0909049513020943 PMC3795544 24121328

[bb20] Nerad, B. A. & Shlichta, P. J. (1986). *J. Cryst. Growth*, **75**, 591–608.

[bb21] Ng, J. D., Baird, J. K., Coates, L., Garcia-Ruiz, J. M., Hodge, T. A. & Huang, S. (2015). *Acta Cryst.* F**71**, 358–370. 10.1107/S2053230X15005348 PMC4388167 25849493

[bb22] Niimura, N. & Podjarny, A. (2011). *Neutron Protein Crystallography*, pp. 50–74. Oxford University Press.

[bb23] Ostwald, W. (1897). *Z. Phys. Chem.* **22**, 289–330.

[bb24] Rupp, B. (2015). *Acta Cryst.* F**71**, 247–260. 10.1107/S2053230X1500374X PMC4356298 25760697

[bb25] Saridakis, E. & Chayen, N. E. (2000). *Protein Sci.* **9**, 755–757. 10.1110/ps.9.4.755 PMC2144611 10794418

[bb26] Schaffner, I., Mlynek, G., Flego, N., Pühringer, D., Libiseller-Egger, J., Coates, L., Hofbauer, S., Bellei, M., Furtmüller, P. G., Battistuzzi, G., Smulevich, G., Djinović-Carugo, K. & Obinger, C. (2017). *ACS Catal.* **7**, 7962–7976. 10.1021/acscatal.7b01749 PMC5678291 29142780

[bb27] Tanaka, H., Yoshizaki, I., Takahashi, S., Yamanaka, M., Fukuyama, S., Sato, M., Sano, S., Motohara, M., Kobayashi, T., Yoshitomi, S. & Tanaka, T. (2006). *Microgravity Sci. Technol.* **18**, 91–94.

[bb28] Thaller, C., Eichele, G., Weaver, L. H., Wilson, E., Karlsson, R. & Jansonius, J. N. (1985). *Methods Enzymol.* **114**, 132–135. 10.1016/0076-6879(85)14011-5 4079759

[bb29] Yoshizaki, I., Nakamura, H., Fukuyama, S., Komatsu, H. & Yoda, S. (2002). *Int. J. Micrograv. Sci. Appl.* **19**, 30–33.

[bb30] Zeppezauer, M. (1971). *Methods Enzymol.* **22**, 253–266.

